# Isolation and diversity of sediment bacteria in the hypersaline aiding lake, China

**DOI:** 10.1371/journal.pone.0236006

**Published:** 2020-07-10

**Authors:** Tong-Wei Guan, Yi-Jin Lin, Meng-Ying Ou, Ke-Bao Chen

**Affiliations:** Institute of Microbiology, Xihua University, Chengdu, P.R. China; Universidade de Coimbra, PORTUGAL

## Abstract

Halophiles are relatively unexplored as potential sources of novel species. However, little is known about the culturable bacterial diversity thrive in hypersaline lakes. In this work, a total of 343 bacteria from sediment samples of Aiding Lake, China, were isolated using nine different media supplemented with 5% or 15% (w/v) NaCl. The number of species and genera of bacteria recovered from the different media varied, indicating the need to optimize the isolation conditions. The results showed an unexpected level of bacterial diversity, with four phyla (*Actinobacteria*, *Firmicutes*, *Proteobacteria*, and *Rhodothermaeota*), fourteen orders (*Actinopolysporales*, *Alteromonadales*, *Bacillales*, *Balneolales*, *Chromatiales*, *Glycomycetales*, *Jiangellales*, *Micrococcales*, *Micromonosporales*, *Oceanospirillales*, *Pseudonocardiales*, *Rhizobiales*, *Streptomycetales*, and *Streptosporangiales*), including 17 families, 43 genera (including two novel genera), and 71 species (including four novel species). The predominant phyla included Actinobacteria and Firmicutes and the predominant genera included *Actinopolyspora*, *Gracilibacillus*, *Halomonas*, *Nocardiopsis*, and *Streptomyces*. To our knowledge, this is the first time that members of phylum *Rhodothermaeota* were identified in sediment samples from a salt lake.

## 1 Introduction

Halophiles thrive in hypersaline niches and have potential applications in biotechnology [1, 2]. Microbial diversity in most hypersaline environments is often studied using culture-dependent and -independent methods [3–7]. Previous studies have shown that the taxonomic diversity of microbial populations in terrestrial saline and hypersaline environments is relatively low [8, 9]. Halophilic microbial communities vary with season [10], and in general, microbial diversity decreases with increased salinity [11, 12]. Hypersaline lakes are considered extreme environments for microbial life. A variety of salt lakes have been surveyed for bacterial diversity such as Chaka Lake in China, Chott El Jerid Lake in Tunisia, Meyghan Lake in Iran, Keke Lake in China, and Great Salt Lake in the United States [5, 13–16]. In addition, groups of novel halophilic or halotolerant bacteria in salt lakes have been described using culture-dependent methods: *Actinopolyspora lacussalsi* sp. nov., *Amycolatopsis halophila* sp. nov., *Brevibacterium salitolerans* sp. nov., *Halomonas xiaochaidanensis* sp. nov., *Paracoccus halotolerans* sp. nov., and *Salibacterium nitratireducens* sp. nov. of phyla *Actinobacteria*, *Firmicutes*, or *Proteobacteria* [17–22]. Despite these previous studies, our understanding of bacterial diversity in hypersaline lakes remains limited, particularly in athalassohaline lakes at low elevations. Aiding Lake represents an ideal site for studying halophilic or halotolerant bacteria in a hypersaline lake. The salt lake is located on the Turpan Basin and surround by the Gobi desert in Xinjiang province, China. In fact, our current understanding of the bacterial diversity in the Aiding Lake using a culture-dependent method is limited. To our knowledge, this is the first attempt to comprehensively characterize the bacterial diversity in dry salt lake sediments. The aim of the study was to investigate the bacterial diversity and to mine novel bacterial species from Aiding Lake.

## 2 Materials and methods

### 2.1 Site description and sample collection

Aiding Lake is a dry salt lake located on the Turpan Basin in Xinjiang Province, China (S1 Fig), with an elevation of 154 m–293 m below sea level. Aiding Lake covers an area of about 60 km^2^ and is a closed ecosystem, without the influx of perennial rivers. Three soil samples, namely, S1 (89°21′98″E, 42°40′42″N), S2 (89°16'6″E, 42°38'55″N), and S3 (89°20'26″E, 42°41'53″N) were collected from the lake sediments, respectively. Soil samples temperature are 23.6°C-25.1°C. Three sediment samples at different sites were collected from a same depth of 1 to 30 cm in mid-July of 2012. The distance between two sample points was greater than 5 km. Samples were stored at 4°C in the field and immediately transported to the laboratory. The pH was measured with portable meters after the sediments were resuspended in distilled water. The concentrations of major cations and trace elements in the dissolved sediments were measured according to Yakimov et al. (2002) [23].

### 2.2 Isolation of microorganisms

Three sediment samples were selected for cultivation of bacteria. To isolate halophilic and/or halotolerant bacteria, the sediments (10 g wet weight) were dispersed into 90 mL of sterilized NaCl brine (5% or 15%, w/v) and incubated at 37°C for 60 min with shaking at 200 rpm. The resulting slurry was then serially diluted with sterilized NaCl brine (5% or 15%, w/v). Aliquots (0.1 mL) of each dilution were spread onto Petri dishes using nine media (Table 1) for the isolation of bacteria. The no. of colonies on each kind of medium plate was calculated by three repeats. All agar plates were supplemented with 5% or 15% (w/v) NaCl. To suppress the growth of nonbacterial fungi, the solidified media were supplemented with nystatin (50 mg·L^-1^). The Petri dishes were incubated at 37°C for one to six weeks. Based on size and color, colonies were picked and further purified on inorganic salts-starch agar [24] or TSA supplemented with 5% or 15% (w/v) NaCl, and as glycerol suspension (20%, v/v) at -20°C or as lyophilized cells for long-term storage at -4°C.

**Table 1:** Compositions of the nine different media used for the isolation of bacteria from Aiding Lake samples.

Medium	Composition	Reference
A	Inorganic salts-starch agar (ISP 4)	Shirling and Gottlieb 1966
B	Casein hydrolysate acid-starch agar: starch 5.0 g, casein hydrolysate acid 0.5 g, KNO_3_ 0.5 g, Aspartic acid 0.1g, CaCO_3_, 0.3 g, K_2_HPO_4_ 0.5 g, MgCl_2_ 0.2 g, FeSO_4_·7H_2_O 10 mg, agar 18 g	This study
C	Microcrystalline cellulose-proline agar: microcrystalline cellulose 2.0 g, proline 0.5 g, arginine 0.1 g, KCl 10 g, (NH_4_)_2_SO_4_ 1.0 g, K_2_HPO_4_ 0.2 g, CaCO_3_ 0.02 g, MgSO_4_ · 7H_2_O 2 g, FeSO_4_ ·7H_2_O 10 mg, MnCl_2_·4H_2_O 1mg, agar 18 g	This study
D	Glycerin-asparagine agar: glycerin 3g, asparagine 1g, C_3_H_3_NaO_3_ 0.5g, MgSO_4_· 7H_2_O 2 g, FeSO_4_·7H_2_O 10 mg, ZnSO_4_·7H_2_O 1 mg, VB1 0.1 mg, VB6 0.05 mg, biotin 0.2mg, agar 18 g	This study
E	Yeast extract-casamino acids agar: yeast extract 3g, Casamino acids 2g, Sodium glutamate 1g, Trisodium citrate 1g, MgSO_4_·7H_2_O 5g, CaCl_2_·2H_2_O 1g, KCl 3g, FeCl_2_·4H_2_O 0.2mg, MnCl_2_·4H_2_O 0.2mg, agar, 18 g	This study
F	Stachyose tetrahydrate-Alanine agar: stachyose tetrahydrate 5g, alanine 2g, KNO_3_ 0.2g, CaCO_3_ 0.02g, MgSO_4_.7H_2_O 0.05g, KCl 20g, FeSO_4_·7H_2_O 10 mg, MgCl_2_ 20g, agar 18 g	This study
G	Microcrystalline cellulose-sorbitol agar: microcrystalline cellulose 10g, sorbitol 2g, Beta-Cyclodextrin 1g, MgSO_4_·7H_2_O 0.1g, CaCO_3_ 0.5g, FeSO_4_ 0.01g, KCl 20g, MgCl_2_ 10g, agar 18 g	This study
H	Yeast extract-fish peptone agar: yeast extract 1g, fish peptone 0.5g, NH_4_Cl 0.5g, MgSO_4_.7H_2_O 20g, MgCl_2_·6H_2_O 15g, KCl 5g, sodium pyruvate 1g, K_2_HPO_4_ 0.3g, CaCl_2_·2H_2_O 0.2g, agar 18 g	This study
I	Yeast extract-glycerin agar: yeast extract 10g, Glycerin 0.5g, peptone 0.5g, (NH_4_)NO_3_ 0.1g, MgCl_2_ 5g, Na_2_SO_4_ 3g, yeast KCl 1g, NaHCO_3_ 2g, KBr 0.05g, SrCl_2_ 0.01g, Na_2_SiO_3_ 0.001g, agar 18 g	This study

### 2.3 Identification of bacteria

Isolated strains were subjected to 16S rRNA gene sequence analysis for precise genus and species identification. Genomic DNA was extracted from each isolate, and the 16S rRNA gene sequence was amplified as described by Li et al. (2007) [25] with primers PA (5'-CAGAGTTTGATCCTGGCT-3') and PB (5'-AGG AGGTGATCCAGCCGC A-3'), or the primers 27F (5'-AGAGTTTGATCMTGGCTCAG-3') and 1492R (5'-GGTTACCTTGTT ACGACTT-3'). PCR products were purified using a PCR purification kit (Sangon, Shanghai, China). The almost-complete 16S rRNA gene sequence (about 1450bp) of isolated strains was obtained. Multiple alignments with sequences of the most closely related recognized species and calculations of levels of sequence similarity were conducted using EzBioCloud server [26]. Phylogenetic analysis was performed using the software package MEGA version 6.0 [27]. Phylogenetic trees were constructed according to the neighbor-joining method [28]. Evolutionary distance matrices were generated as described by Kimura (1980) [29]. The topology of the phylogenetic tree was evaluated using the bootstrap resampling method of Felsenstein (1985) [30] with 1000 replicates. DNA-DNA relatedness values were determined using the fluorometric microwell method [31]. The identities of these organisms were determined based on nearly full-length 16S rRNA gene sequence analysis. The sequences of 100% identity were clustered into one species.

### 2.4 Spearman correlation analysis

Pearson’s test was performed to reveal the correlations between physicochemical properties and bacterial genera using SPSS Statistics19.0.

### 2.5 Nucleotide sequence accession numbers

The sequences of the bacterial isolates reported in this study have been deposited to GenBank (Accession no. MK818765- MK818834, MK296404).

## 3 Results

### 3.1 Sediment geochemistry

Physicochemical parameters were distinct among the three sediment samples (Table 2). In the S1, S2, and S3 samples, Na^+^ concentrations ranged from 26.37 g/Kg to 83.93 g/Kg and Cl^-^ concentrations ranged from 33.28 g/Kg to 389.11 g/Kg, which are typical of chloride-type environments. The pH of the sediment samples ranged from 7.6 to 8.3, indicating a slightly alkaline environment. In sample S3, ionic composition (e.g., Mg^2+^, Na^+^, K^+^, Mn^2+^, and Cl^-^) was significantly lower than the other samples. The other physicochemical properties of the samples are presented in Table 2.

**Table 2:** Physicochemical properties of the sediments from the three sample sites in Aiding Lake.

Site	pH	Ion concentration (g Kg^-1^)								
		Ca^2+^	Mg^2+^	Fe^2+^	Na^+^	K^+^	Mn^2+^	Cl^-^	SO_4_^2-^	HCO_3_^-^
S1	8.3	6.54	1.93	63ppm	83.93	0.33	23ppm	389.11	85.25	0.06
S2	8.1	0.24	2.43	102ppm	38.29	0.25	9ppm	45.38	6.42	0.13
S3	7.6	4.25	0.61	82ppm	26.37	0.16	2ppm	33.28	23.52	0.41

ppm, parts per million.

### 3.2 Diversity of sediment bacteria

**Fig 1:**
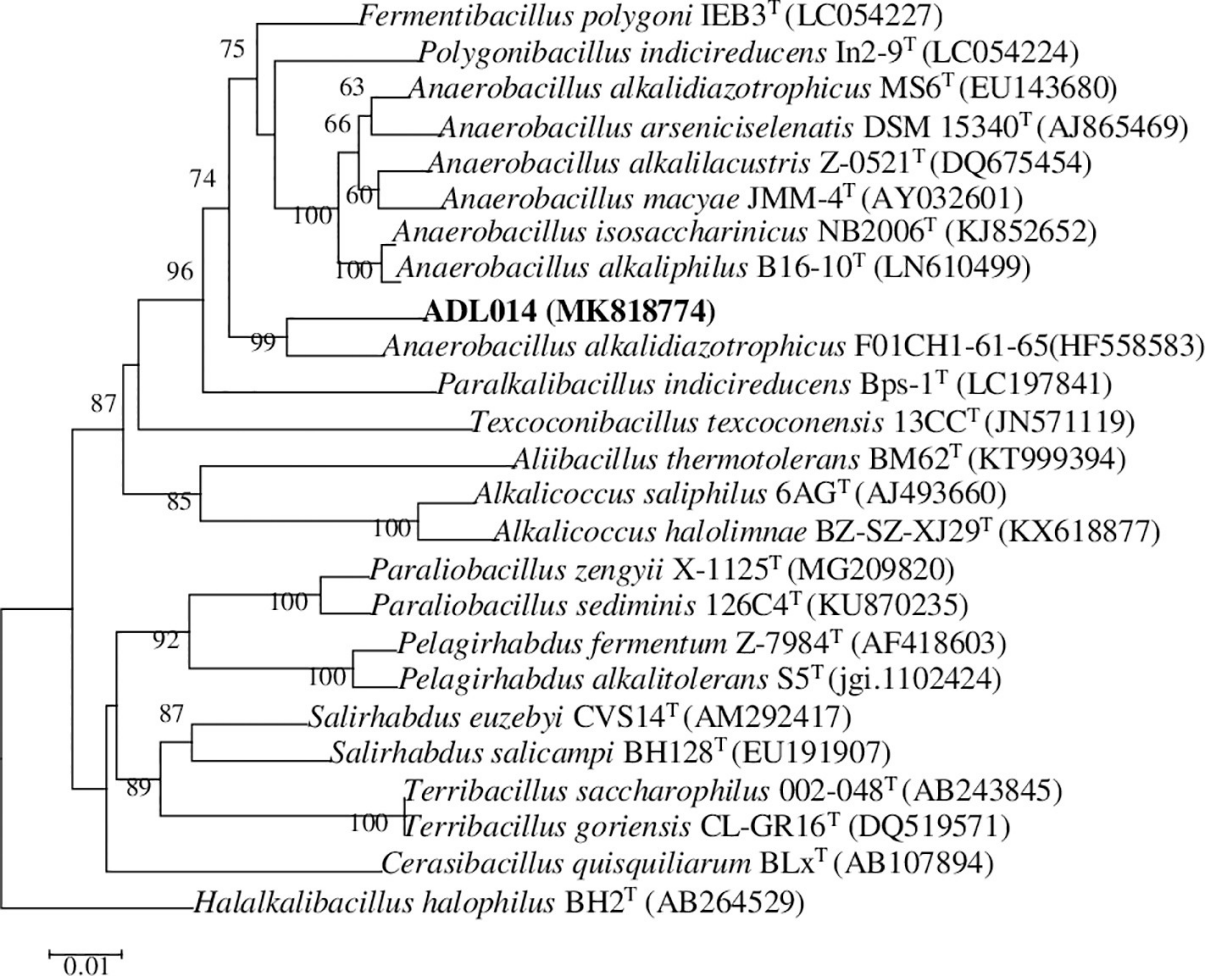
Phylogenetic tree of strain ADL014 and its near neighbors calculated from 16S rRNA gene sequences using Kimura’s evolutionary distance method (Kimura, 1980) and the neighbor-joining method of Saitou and Nei (1987). Bar, 0.01 nucleotide substitutions per site.

According to the analysis of sequencing results, many of isolated strains had exactly the same 16S rRNA sequence. 343 isolated strains belong to 71 different bacterial species after merging the duplicated strains (Table 3). The percentages of 16S rRNA gene sequence similarities (91.14% to 100%) of these isolates to the closest type strains are presented in Table 3. As the results indicated, most of the strains exhibited > 97% similarity to other published type species. While the similarity of three strains (ADL013, ADL014, and ADL023) were less than 97%, which represented three different novel species (Table 3). It is generally accepted that organisms displaying 16S rRNA gene sequence similarity values of 97% or less belong to different species [32]. For example, strain ADL014 shared 96.51% similarity with *Anaerobacillus alkalidiazotrophicus* F01CH1-61-65, and DNA-DNA hybridization experiments from the two strains revealed that levels of DNA-DNA relatedness were 36.8± 4.3%. Sequence analysis indicated that strain ADL014 formed a distinct lineage within the genus *Anaerobacillus* and always had the closest phylogenetic affinity to members of the genus *Anaerobacillus* (Fig 1). Phylogenetic reconstruction also indicated that strain ADL014 could represent a novel species.

**Table 3:** Bacteria isolated using two different salinity from sediments of Aiding Lake, with the similarity values for 16S rRNA gene sequences.

Isolate	Salinity	Closest cultivated species (GenBank accession no.)	Similarity (%)	No. of isolates
ADL001	15%	*Actinopolyspora alba* YIM 90480 (GQ480940)	98.50	3
ADL003	15%	*Actinopolyspora mortivallis* DSM 44261(NR_043996.1)	98.80	1
ADL004	15%	*Actinopolyspora halophila* DSM 43834 (AQUI01000002)	100	9
ADL007	15%	*Actinopolyspora xinjiangensis* DSM 46732 (jgi.1055186)	99.71	13
ADL008	15%	*Aidingimonas halophila* DSM 19219 (jgi.1107932)	97.76	2
ADL009	15%	*Aliifodinibius salicampi* KHM44 (LC198077)	99.58	5
ADL011	15%	*Alteribacillus alkaliphilus* JC229 (HG799487)	98.12	1
ADL012	5%	*Alteribacillus bidgolensis* IBRC-M10614 (jgi.1071278)	99.86	7
ADL013	15%	*Alteribacillus persepolensis* HS136 (FM244839)	94.71	1
ADL014	5%	*Anaerobacillus uncultured bacterium* F01CH1-61-65 (HF558583)	96.51	1
ADL015	15%	*Aquibacillus albus* YIM 93624 (JQ680032)	100	9
ADL017	5%	*Aquibacillus koreensis* BH30097 (AY616012)	97.28	1
ADL018	15%	*Aquisalimonas halophila* YIM 95345 (KC577145)	100	12
ADL019	15%	*Bacillus halmapalus* DSM 8723 (KV917375)	99.29	6
ADL020	15%	*Bacillus salarius* BH169 (AY667494)	98.98	3
ADL021	5%	*Bacillus swezeyi* NRRL B-41294 (MRBK01000096)	99.79	6
ADL022	15%	*Filobacillus milosensis* DSM 13259 (AJ238042)	99.53	4
ADL023	15%	*Caldalkalibacillus uzonensis* JW/WZ-YB58 (DQ221694)	91.14	1
ADL024	5%	*Glycomyces xiaoerkulensis* TRM 41368 (MF669725)	99.72	2
ADL026	15%	*Gracilibacillus bigeumensis* BH097 (EF520006)	99.57	16
ADL027	5%/15%	*Gracilibacillus orientalis* XH-63 (AM040716)	98.36	2
ADL028	15%	*Gracilibacillus saliphilus* YIM 91119 (EU784646)	99.58	8
ADL029	5%/15%	*Gracilibacillus thailandensis* TP2-8 (FJ182214)	97.86	6
ADL030	15%	*Gracilibacillus ureilyticus* MF38 (EU709020)	97.95	1
ADL031	15%	*Haloactinospora alba* YIM 90648 (DQ923130)	99.44	3
ADL032	15%	*Halobacillus dabanensis* D-8 (AY351395)	99.11	5
ADL033	5%	*Halobacillus yeomjeoni* MSS-402 (AY881246)	98.52	3
ADL034	15%	*Haloechinothrix halophila* YIM 93223 (KI632509)	97.93	2
ADL036	5%	*Halomonas arcis* AJ282 (EF144147)	99	17
ADL037	15%	*Halomonas lutea* DSM 23508 (ARKK01000003)	100	4
ADL038	5%/15%	*Halomonas xinjiangensis* TRM 0175 (JPZL01000008)	99.5	21
ADL040	5%/15%	*Jeotgalibacillus terrae* JSM 081008 (FJ527421)	99.15	4
ADL041	5%	*Kocuria assamensis* S9-65 (HQ018931)	99.64	1
ADL042	5%	*Kocuria palustris* DSM 11925 (Y16263)	99.72	1
ADL044	5%	*Longimycelium tulufanense* TRM 46004 (HQ229000)	100	5
ADL045	5%	*Marinactinospora thermotolerans* DSM 45154 (FUWS01000037)	99.29	6
ADL047	5%	*Marinobacter guineae* M3B (AM503093)	98.59	3
ADL048	5%/15%	*Marinobacter lacisalsi* FP2.5 (EU047505)	98.9	5
ADL049	15%	*Marinococcus luteus* DSM 23126 (jgi.1089306)	100	11
ADL050	5%	*Micromonospora andamanensis* SP03-05 (JX524154)	99.05	2
ADL053	5%	*Micromonospora halotolerans* CR18(NR_132303.1)	100	3
ADL054	5%	*Myceligenerans salitolerans* XHU 5031 (JX316007)	100	2
ADL055	15%	*Nesterenkonia halophila* YIM 70179 (AY820953)	99	2
ADL056	5%	*Nitratireductor shengliensis* 110399 (KC222645)	97.58	3
ADL057	5%	*Nocardiopsis aegyptia* DSM 44442 (AJ539401)	99.43	7
ADL060	5%	*Nocardiopsis mwathae* No.156 (KF976731)	98.72	3
ADL061	15%	*Nocardiopsis rosea* YIM 90094 (AY619713)	99.27	6
ADL063	5%	*Nocardiopsis sinuspersici* HM6 (EU410476)	98.8	1
ADL065	5%	*Ornithinibacillus scapharcae* TW25 (AEWH01000025)	98.52	1
ADL066	15%	*Phytoactinopolyspora halotolerans* YIM 96448 (KY979511)	100	3
ADL067	15%	*Piscibacillus halophilus* HS224 (FM864227)	99.01	5
ADL068	5%	*Planococcus salinarum* DSM 23820 (MBQG01000128)	98.82	2
ADL069	15%	*Pontibacillus marinus* BH030004 (AVPF01000156)	99.12	9
ADL070	5%	*Prauserella marina* CGMCC 4.5506 (jgi.1085010)	97.3	1
ADL071	15%	*Saccharomonospora azurea* NA-128 (AGIU02000033)	100	6
ADL073	15%	*Saccharomonospora xiaoerkulensis* TRM 41495 (KU511278)	99.72	1
ADL075	15%	*Saccharopolyspora lacisalsi* TRM 40133 (JF411070)	100	5
ADL076	15%	*Salinicoccus luteus* YIM 70202 (DQ352839)	100	9
ADL078	5%	*Salinifilum aidingensis* TRM 46074 (JX193858)	99.93	4
ADL079	5%	*Sediminibacillus halophilus* EN8d (AM905297)	100	11
ADL080	15%	*Sinobaca qinghaiensis* YIM 70212 (DQ168584)	100	5
ADL082	5%	*Streptomyces aidingensis* TRM46012 (HQ286045)	100	6
ADL083	5%	*Streptomyces ambofaciens* ATCC 23877 (CP012382)	99.31	2
ADL084	5%	*Streptomyces asenjonii* KNN 35.1b (LT621750)	98.91	3
ADL086	5%	*Streptomyces coelicoflavus* NBRC 15399 (AB184650)	99.79	3
ADL087	5%	*Streptomyces fukangensis* EGI 80050 (KF040416)	98.6	2
ADL088	5%	*Streptomyces griseoincarnatus* LMG 19316 (AJ781321)	99.93	7
ADL090	5%	*Streptomyces xinghaiensis* S187 (CP023202)	99.93	4
ADL091	5%/15%	*Virgibacillus sediminis* YIM kkny3 (AY121430)	99.65	11
ADL092	5%	*Zhihengliuella somnathii* JG 03 (EU937748)	99.17	2
XHU5135	15%	*Aidingimonas halophila* BH017 (EU191906)	97.52	1

**Fig 2:**
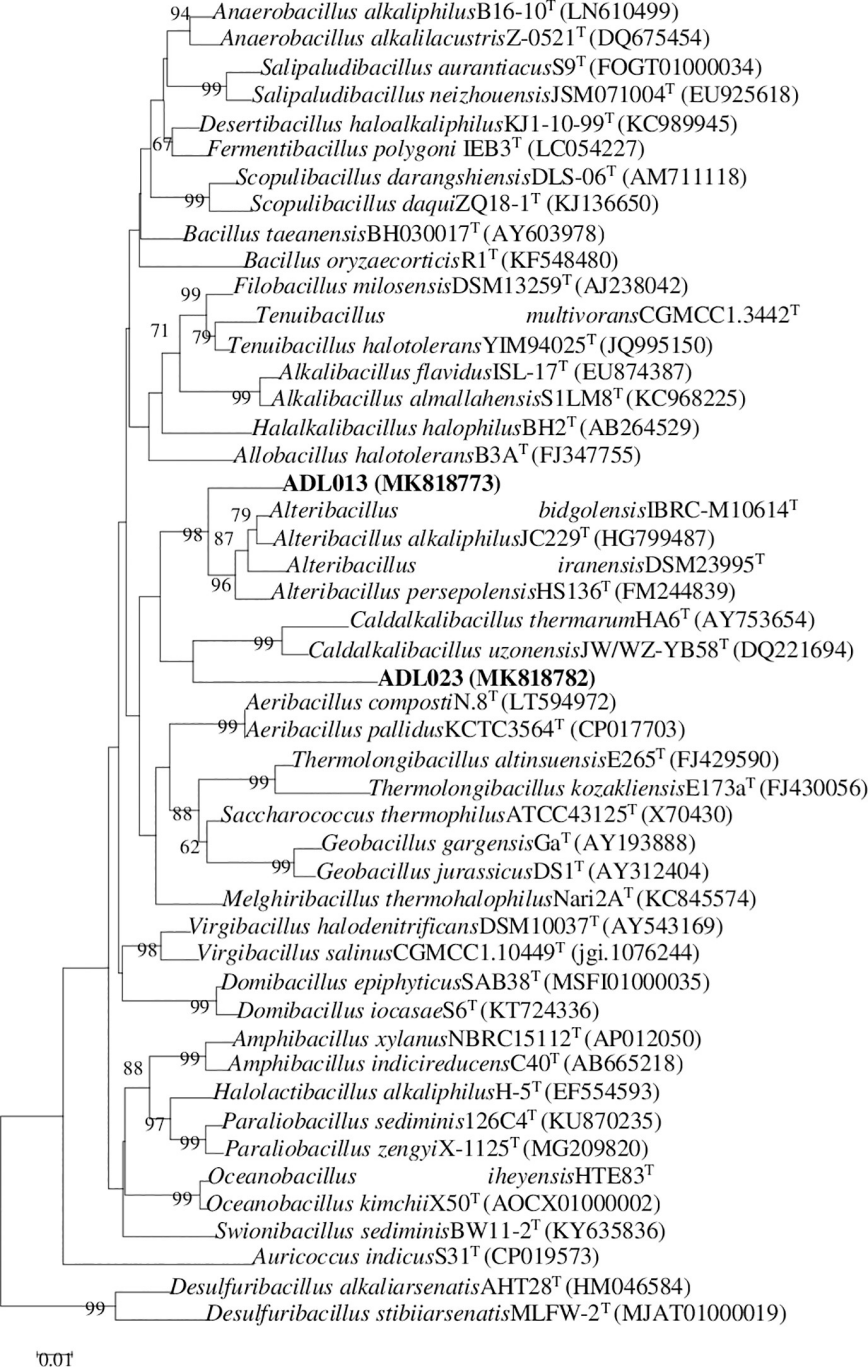
Phylogenetic dendrogram for taxa of the family *Bacillaceae* reconstructed using the neighbor-joining method based on almost complete 16S rRNA gene sequences to display the taxonomic position of strain ADL013 or strain ADL023. Numbers at nodes indicate levels of bootstrap support (%) based on neighbor-joining analysis of 1000 resampled datasets; only values above 50% are shown. Bar, 0.01 nucleotide substitutions per site.

**Fig 3:**
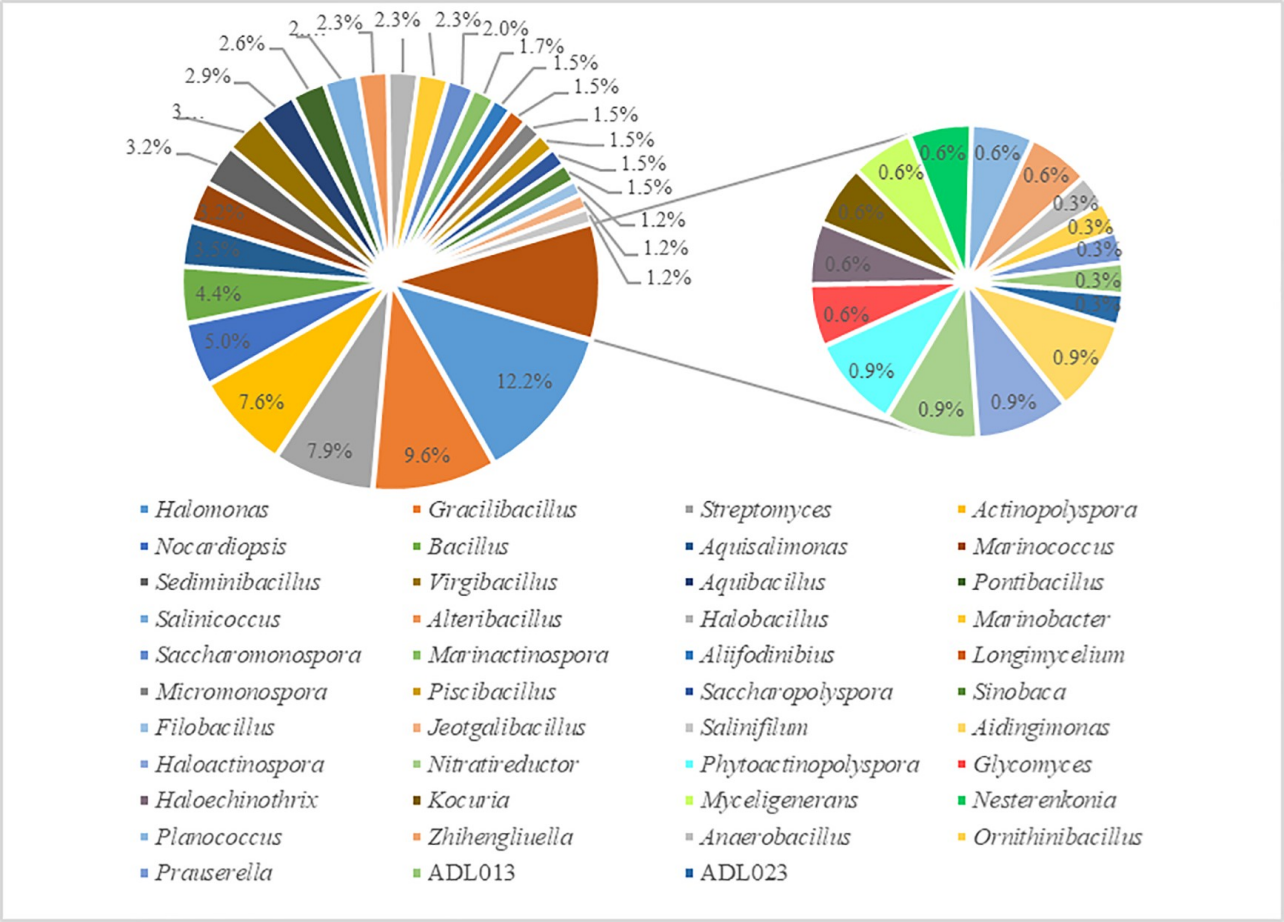
Percentage of isolated strains in each genus.

These halophilic or halotolerant bacteria were compared to those deposited in the public database (EzBioCloud, https://www.ezbiocloud.net/identify). The bacteria isolated in this study displayed considerable diversity. The predominant phyla were *Firmicutes* (149 strains, 43.4%) and *Actinobacteria* (121 strains, 35.3%). The other bacterial isolates belonged to phyla *Rhodothermaeota* (5 strains, 1.5%) and *Proteobacteria* (68 strains, 19.8%). The isolates were distributed among 14 orders, namely, *Actinopolysporales* (26 strains), *Alteromonadales* (8 strains), *Bacillaceae* (149 strains), *Balneolales* (5 strains), *Chromatiales* (12 strains), *Glycomycetales* (2 strains), *Jiangellales* (3 strains), *Micrococcales* (8 strains), *Micromonosporineae* (5 strains), *Oceanospirillales* (45 strains), *Pseudonocardiales* (11 strains), *Rhizobiales* (3 strains), *Streptomycetales* (27 strains), and *Streptosporangiales* (26 strains), including 41 known genera (Tables 3 and 4). Other organisms (ADL013 and ADL023) could not be accurately identified to the genus level because of the lower homology (Table 3). Strain ADL013 exhibited 94.71% similarity to the 16S rRNA gene sequence of *Alteribacillus persepolensis* HS136, and the hybridization values of 11.3±2.1% to each other; and strain ADL023 exhibited 91.14% similarity to the 16S rRNA gene sequence of *Caldalkalibacillus uzonensis* JW/WZ-YB58, and the hybridization values of 8.3±1.7% to each other. Phylogenetic analysis also showed that strain ADL013 and ADL023 can be distinguished from representatives of genera in the family *Bacillaceae*, and two strains formed a distinct lineage within family *Bacillaceae*, respectively (Fig 2). Meanwhile, two strains had such low degrees of sequence similarity, suggesting that these may represent two novel genera of *Bacillaceae*. In the study, *Halomonas* (42 strains, 12.2%), *Gracilibacillus* (33 strains, 9.6%), *Streptomyces* (27 strains, 7.9%), *Actinopolyspora* (26 strains, 7.6%), *Nocardiopsis* (17 strains, 5.0%), *Bacillus* (12 strains, 4.4%), *Aquisalimonas* (12 strains, 3.5%), *Marinococcus* (11 strains, 3.2%), *Virgibacillus* (11 strains, 3.2%), and *Sediminibacillus* (11 strains, 3.2%) were some dominant group in sediment samples of Aiding Lake. The number of microorganisms in each of the other genus is relatively small (Table 3; Fig 3). For example, *Anaerobacillus*, *Prauserella*, and *Ornithinibacillus* include only one strain, respectively. To isolate halophilic or halotolerant bacteria in the sediments of Aiding Lake, the agar plates were supplemented with 5% or 15% (w/v) NaCl. Approximately 141 strains isolated from these media with 5% NaCl belonged to 25 different genera, and 202 strains isolated from the media using 15% NaCl belonged to 23 different genera (Table 3).

**Table 4:** Statistical analyses of the relationships between the taxa of the bacterial strains and the nine different media.

Medium	No. of isolates	Isolate taxon			
		Phylum	Order	Family	Genus
A	27	*Actinobacteria*	*Micromonosporales*	*Micromonosporaceae*	*Micromonospora*
			*Pseudonocardiales*	*Pseudonocardiaceae*	*Prauserella*
			*Streptomycetales*	*Streptomycetaceae*	*Streptomyces*
			*Streptosporangiales*	*Nocardiopsaceae*	*Nocardiopsis*
B	24	*Actinobacteria*	*Actinopolysporales*	*Actinopolysporaceae*	*Actinopolyspora*
			*Micromonosporales*	*Micromonosporaceae*	*Micromonospora*
			*Pseudonocardiales*	*Pseudonocardiaceae*	*Saccharopolyspora*
			*Streptomycetales*	*Streptomycetaceae*	*Streptomyces*
		*Firmicutes*	*Bacillales*	*Bacillaceae*	*Halobacillus*
		*Proteobacteria*	*Alteromonadales*	*Planococcaceae*	*Planococcus*
				*Alteromonadaceae*	*Marinobacter*
C	57	*Actinobacteria*	*Actinopolysporales*	*Actinopolysporaceae*	*Actinopolyspora*
			*Glycomycetales*	*Glycomycetaceae*	*Glycomyces*
			*Micrococcales*	*Micrococcaceae*	*Kocuria*
					*Nesterenkonia*
			*Pseudonocardiales*	*Promicromonosporaceae*	*Myceligenerans*
			*Streptosporangiales*	*Pseudonocardiaceae*	*Saccharomonospora*
				*Nocardiopsaceae*	*Haloactinospora*
		*Firmicutes*	*Bacillales*	*Bacillaceae*	*Salinifilum*
					*Anaerobacillus*
					*Jeotgalibacillus*
					*Pontibacillus*
D	25	*Actinobacteria*	*Actinopolysporales*	*Actinopolysporaceae*	*Actinopolyspora*
			*Pseudonocardiales*	*Pseudonocardiaceae*	*Longimycelium*
			*Streptomycetales*	*Streptomycetaceae*	*Streptomyces*
			*Streptosporangiales*	*Nocardiopsaceae*	*Saccharomonospora*
					*Nocardiopsis*
		*Firmicutes*	*Bacillales*	*Bacillaceae*	*Marinococcus*
E	20	*Rhodothermaeota*	*Balneolales*	*Balneolaceae*	*Aliifodinibius*
		*Firmicutes*	*Bacillales*	*Bacillaceae*	*Bacillus*
				*Staphylococcaceae*	*Salinicoccus*
F	57	*Actinobacteria*	*Pseudonocardiales*	*Pseudonocardiaceae*	*Haloechinothrix*
			*Streptomycetales*	*Streptomycetaceae*	*Streptomyces*
			*Streptosporangiales*	*Nocardiopsaceae*	*Marinactinospora*
					*Nocardiopsis*
		*Firmicutes*	*Bacillales*	*Bacillaceae*	*Aquibacillus*
					*Halobacillus*
		*Proteobacteria*	*Alteromonadales*	*Marinobacter family*	*Marinobacter*
			*Oceanospirillales*	*Halomonadaceae*	*Halomonas*
G	95	*Actinobacteria*	*Jiangellales*	*Jiangellaceae*	*Phytoactinopolyspora*
			*Micrococcales*	*Micrococcaceae*	*Kocuria*
					*Zhihengliuella*
			*Streptosporangiales*	*Nocardiopsaceae*	*Nocardiopsis*
		*Firmicutes*	*Bacillales*	*Bacillaceae*	*Bacillus*
					*Aquibacillus*
					*Filobacillus*
					*Gracilibacillus*
					*Ornithinibacillus*
					*Piscibacillus*
					*Virgibacillus*
		*Proteobacteria*	*Chromatiales*	*Nitrococcus family*	*Aquisalimonas*
			*Oceanospirillales*	*Halomonadaceae*	*Halomonas*
H	24	*Firmicutes*	*Bacillales*	*Bacillaceae*	*Alteribacillus*
					*Gracilibacillus*
					ADL023
		*Proteobacteria*	*Rhizobiales*	*Phyllobacteriaceae*	*Nitratireductor*
			*Oceanospirillales*	*Halomonadaceae*	*Aidingimonas*
I	14	*Firmicutes*	*Bacillales*	*Bacillaceae*	*Gracilibacillus*
					*Sinobaca*
					ADL013

### 3.3 Bacterial isolates from different media

To obtain additional bacterial groups, the sediment samples from Aiding Lake were isolated using nine different media (Table 1). Five class of bacteria, namely, *Actinobacteria*, *Bacilli*, *Alphaproteobacteria*, *Gammaproteobacteria*, and *Balneolia*, including 14 orders, 17 families, and 43 genera (including 2 novel genera) were obtained (Table 4). Most of the bacterial groups were isolated using microcrystalline cellulose-sorbitol agar (G), and 13 bacterial genera (*Aquibacillus*, *Aquisalimonas*, *Bacillus*, *Filobacillus*, *Gracilibacillus*, *Halomonas*, *Kocuria*, *Nocardiopsis*, *Ornithinibacillus*, *Phytoactinopolyspora*, *Piscibacillus*, *Virgibacillus*, and *Zhihengliuella*) were isolated. At the same time, microcrystalline cellulose-proline agar (C), stachyose tetrahydrate-alanine agar (F), casein hydrolysate acid-starch agar (B), and glycerin-asparagine agar (D) resulted in relatively efficient isolations for 11 genera (*Actinopolyspora*, *Anaerobacillus*, *Glycomyces*, *Haloactinospora*, *Jeotgalibacillus*, *Kocuria*, *Myceligenerans*, *Nesterenkonia*, *Pontibacillus*, *Saccharomonospora*, and *Salinifilum*), 8 genera (*Aquibacillus*, *Halobacillus*, *Haloechinothrix*, *Halomonas*, *Marinactinospora*, *Marinobacter*, *Nocardiopsis*, and *Streptomyces*), 7 genera (*Actinopolyspora*, *Halobacillus*, *Marinobacter*, *Micromonospora*, *Planococcus*, *Saccharopolyspora*, and *Streptomyces*), and 6 genera (*Actinopolyspora*, *Longimycelium*, *Marinococcus*, *Nocardiopsis*, *Saccharomonospora*, and *Streptomyces*), respectively. Only two known genera (*Gracilibacillus* and *Sinobaca*) and one novel genus (ADL013) were isolated using yeast extract-glycerin agar (I). The results of isolation using the other media are shown in Table 4. From the number of isolated strains, 95, 57, and 57 strains were isolated using media G, F, and C, respectively, and the number of strains isolated from media A, B, D, E, H and I was relatively small (Tables 3 and 4). In addition, the bacterial diversity recovered using different media also varied considerably. Medium G had the best recoverability, with 14 of the total bacterial genera recovered. Media I and E showed the lowest recoverability at the genus level (Table 4). In short, to obtain more bacterial resources, it is also necessary to develop different types of media.

### 3.4 Bacterial isolates from different sediments and Pearson correlation

The number of isolates of bacteria recovered from three sediments (S1, S2, and S3) are different.164, 88 and 91 strains were isolated from sediment samples S1, S2, and S3, respectively (S1 Table). In sediment sample S1, 28 bacterial genera were isolated, while another 27, and 24 bacterial genera were isolated from samples S2 and S3, respectively (S1 Table). Although there was a small difference in the number of genera per sample, there was a large difference in the type of genera per sample (S1 Table). For example, *Haloactinospora*, *Nesterenkonia*, *Phytoactinopolyspora*, *Saccharomonospora*, *Saccharopolyspora*, ADL013, and ADL023 were isolated only from sample S1; *Glycomyces*, *Haloechinothrix*, *Myceligenerans*, *Ornithinibacillus*, and *Prauserella* were isolated only from sample S2; meanwhile, *Anaerobacillus*, *Kocuria*, *Salinifilum*, and *Zhihengliuella* were also isolated only from sample S3 (S1 Table). In addition, *Actinopolyspora*, *Aquibacillus*, *Aquisalimonas*, *Bacillus*, *Gracilibacillus*, *Halomonas*, *Marinococcus*, *Nocardiopsis*, *Salinicoccus*, *Streptomyces*, and *Virgibacillus* were distributed in all three samples (S1 Table). The results showed that the number and diversity of bacteria were different even in different sites from the same salt lake.

Pearson’s correlation analysis revealed that Na^+^, K^+^, Cl^-^ and HCO_3_^-^ were mainly positively correlated with the relative abundances of *Actinopolyspora*, *Gracilibacillus*, *Pontibacillus*, *Aidingimonas*, *Aquisalimonas*, *Halobacillus*, *Bacillus*, *Haloactinospora*, *Nesterenkonia*, *Phytoactinopolyspora*, *Saccharomonospora*, *Saccharopolyspora*, ADL013, ADL023, *Alteribacillus*, and *Nocardiopsis*, respectively (Table 5). Mn^2+^ was mainly positively correlated with the relative abundances of *Aquibacillus*, *Filobacillus*, and *Marinococcus* but negatively correlated with *Micromonospora*. Fe^2+^ was mainly positively correlated with the relative abundances of *Marinactinospora*. SO_4_^2-^ was mainly positively correlated with the relative abundances of *Salinicoccus* but negatively correlated with *Piscibacillus* (Table 5).

**Table 5:** Pearson correlation coefficient (*r*) and *p*-value for isolated bacterial genera. Only correlations with *p* ≤ 0.05 are shown.

Genus	Correlation with	*p*-Value	*r*
*Actinopolyspora*	Na^+^	0.04	0.99
*Aidingimonas*	K^+^	0.02	0.99
*Aliifodinibius*	SO_4_^2-^	0.02	0.99
*Alteribacillus*	HCO_3_^-^	0.04	0.99
*Aquibacillus*	Mn^2+^	0.00	1.00
*Aquisalimonas*	K^+^	0.02	0.99
*Bacillus*	Cl^-^	0.02	1.00
*Filobacillus*	Mn^2+^	0.00	1.00
*Gracilibacillus*	Na^+^	0.04	0.99
*Haloactinospora*	Cl^-^	0.02	1.00
*Halobacillus*	K^+^	0.05	0.99
*Marinactinospora*	Fe^2+^	0.01	–1.00
*Marinococcus*	Mn^2+^	0.00	1.00
*Micromonospora*	Mn^2+^	0.00	–1.00
*Nesterenkonia*	Cl^-^	0.02	1.00
*Nocardiopsis*	pH, HCO_3_^-^	0.04, 0.02	–0.99, 1.00
*Phytoactinopolyspora*	Cl^-^	0.02	1.00
*Piscibacillus*	SO_4_^2-^	0.02	–0.99
*Pontibacillus*	Na^+^, Cl^-^,	0.05, 0.05	0.99, 0.99
*Saccharomonospora*	Cl^-^	0.02	1.00
*Saccharopolyspora*	Cl^-^	0.02	1.00
*Salinicoccus*	SO_4_^2-^	0.02	0.99
ADL013	Cl^-^	0.02	1.00
ADL023	Cl^-^	0.02	1.00

## 4 Discussion

Bacterial diversity in Aiding Lake was based on the 16S rRNA gene sequences, which was relatively higher than other salt lakes at the genus level. For example, sequencing of 16S rRNA genes indicated the presence of members of bacterial genera *Bacillus*, *Halomonas*, *Pseudomonas*, *Exiguobacterium*, *Vibrio*, *Paenibacillus*, and *Planococcus* in the salt lake La Sal del Rey, in extreme South Texas (USA) [33]. Previous studies also have shown that bacterial diversity in other saline lake ecosystems were mainly composed of the bacteria (including 16 genera: *Bacillus*, *Chromohalobacter*, *Gracilibacillus*, *Halobacillus*, *Halolactibacillus*, *Halomonas*, *Halovibrio*, *Idiomarina*, *Oceanobacillus*, *Piscibacillus*, *Salicola*, *Salimicrobium*, *Salinicoccus*, *Staphylococcus*, *Thalassobacillus*, and *Virgibacillus*) [34, 35]. Notably, some rare genera from the sediments of Aiding Lake such as *Aidingimonas*, *Aliifodinibius*, *Filobacillus*, *Haloechinothrix*, *Jeotgalibacillus*, *Longimycelium*, *Myceligenerans*, *Ornithinibacillus*, *Phytoactinopolyspora*, and *Piscibacillus* were discovered in the present study. In addition, phylum *Rhodothermaeota* was detected for the first time in sediment samples from a salt lake.

The richness and diversity of actinobacteria isolated from Aiding Lake in the present study were relatively high, consisting of eight orders (*Actinopolysporales*, *Glycomycetales*, *Jiangellales*, *Micrococcales*, *Micromonosporales*, *Pseudonocardiales*, *Streptomycetales* and *Streptosporangiales*). Wu et al. (2008) have found six actinobacterial orders using pure culture in salt lake, hypersaline spring, and salt mine of China, and these orders were *Actinopolysporales*, *Glycomycetales*, *Micrococcales*, *Pseudonocardiales*, *Streptomycetales*, and *Streptosporangiales* [36]. Our results suggested that the diversity of actinobacterial 16S rRNA gene sequences of strains from Aiding Lake were more diverse at the genus level than those reported in saline environment. For examples, twelve genera, namely *Actinomadura*, *Actinomycetospora*, *Microbispora*, *Micromonospora*, *Mycobaterium*, *Nocardia*, *Nocardiopsis*, *Pseudonocardia*, *Saccharopolyspora*, *Sphaerosporangium*, *Streptomyces*, and *Streptosporangium* were isolated from marine sponges in Florida, USA [37], and ten genera, including *Actinotalea*, *Arthrobacter*, *Brachybacterium*, *Brevibacterium*, *Kocuria*, *Kytococcus*, *Microbacterium*, *Micrococcus*, *Mycobacterium*, and *Pseudonocardia* from Arctic marine sediments were obtained by culture-dependent approaches [38]. Atika et al. isolated fifty-two halophilic actinomycetes belonging to the *Actinopolyspora*, *Nocardiopsis*, *Saccharomonospora*, *Saccharopolyspora*, and *Streptomonospora* genera from Saharan saline soils of Algeria [39]. Ronoh et al. identified four genera (*Dietzia*, *Microbacterium*, *Nocardia*, and *Rhodococcus*) of actinobacteria from Lake Magadi, Kenya [40]. However, 18 genera, namely *Actinopolyspora*, *Glycomyces*, *Haloactinospora*, *Haloechinothrix*, *Kocuria*, *Longimycelium*, *Marinactinospora*, *Micromonospora*, *Myceligenerans*, *Nesterenkonia*, *Nocardiopsis*, *Phytoactinopolyspora*, *Prauserella*, *Saccharomonospora*, *Saccharopolyspora*, *Salinifilum*, *Streptomyces*, and *Zhihengliuella* were isolated in this study. Therefore, bacterial diversity may differ between different sample collection regions and different saline samples. Of course, it is also possible that the diversity of isolated bacteria may vary due to different media or isolated technology.

**Fig 4:**
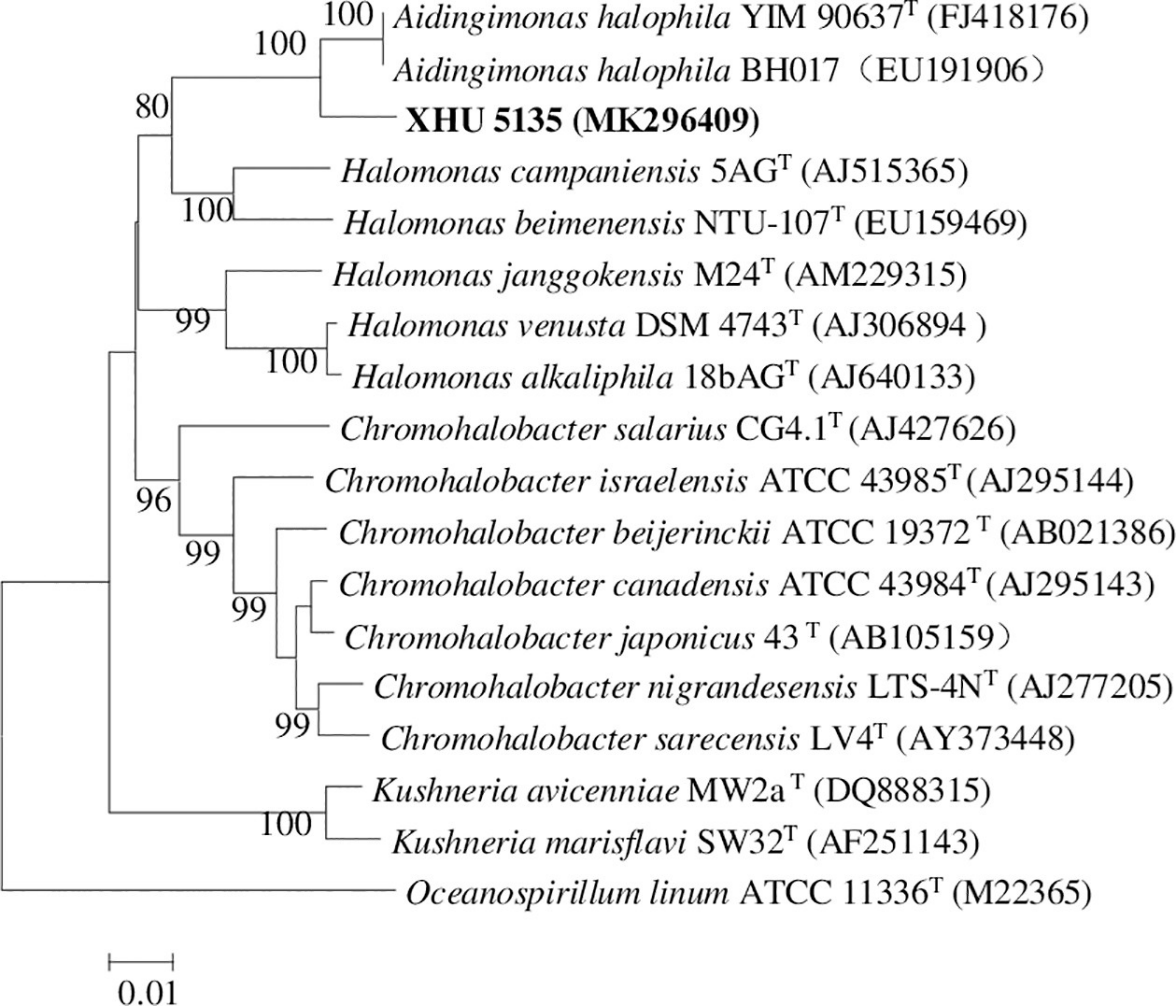
Neighbour-joining tree based on 16S rRNA gene sequences, showing the phylogenetic relationships of the novel isolate XHU 5135 and related taxa. Numbers at nodes are bootstrap percentages based on 1000 replicates. Bar, 0.01 nucleotide substitutions per site.

In addition to strains ADL013, ADL014, and ADL023 are novel species because of low similarity, the 16S rRNA gene sequences of strain XHU5135 showed 97.52% identities to the nearest neighbors, *Aidingimonas halophila* YIM 90637 [41]. Although it showed higher 16S rRNA gene similarities (97.52%) to the closest recognized strains, DNA-DNA hybridization experiments revealed that levels of DNA-DNA relatedness between strain XHU 5135 and *Aidingimonas halophila* YIM 90637 were 23.3± 2.8%. This values is below the 70% cut-off point recommended for recognition of genomic species [42]. Thus, strain XHU 5135 represents a novel species of the genus *Aidingimonas*. The neighbour-joining phylogenetic tree based on 16S rRNA gene sequences of strain XHU 5135 and other related species is shown in Fig 4 with high levels of bootstrap support. At present, we isolated and cultured 71 species (including 4 novel genera) of 43 bacterial genera (including 2 novel genera). To our knowledge, this is the first study to recover such a high diversity of culturable bacteria from salt lake sediments. In addition, *Aidingimonas halophila* YIM 90637 [41], *Streptomyces aidingensis* TRM46012 [43], *Longimycelium tulufanense* TRM 46004 [44], *Salinifilum aidingensis* TRM 46074 [45], and *Gracilibacillus aidingensis* YIM 98001 [46] were also isolated from Aiding Lake, and above strains have been characterized as some novel species. These results indicate that there are potentially unique, novel sources of bacteria in Aiding Lake.

The dry lake has a high NaCl content. However, a large number of bacteria are isolated using the media employed a lower content of NaCl (5%). Possible reasons for such inconsistency could be twofold: (1) bacteria may tolerate a large range of salinity. For example, *Halomonas xinjiangensis* TRM 0175 could grow at 0–20% NaCl [47]; (2) surface layer of the soil samples have a high salinity, while the other parts have a low salinity.

Generally, bacterial diversity in extreme environments is relatively low. Therefore, according to the differences in the utilization of carbon and nitrogen sources by microorganisms, as well as the high repetition rate of microorganisms isolated from conventional media, we designed some media containing rare carbon and nitrogen source (microcrystalline cellulose, glycerin, stachyose tetrahydrate, sorbitol, Beta-Cyclodextrin, casein hydrolysate acid, proline, arginine, asparagine, or alanine) in the hope of mining more novel species and presenting better bacterial diversity. To our satisfaction, an unexpectedly high bacterial diversity was observed from sediment samples from Aiding Lake. Forty-three bacterial genera were identified. The results indicate that it is feasible to use rare carbon and nitrogen source media to mine more species, and some strains might represent a valuable source of new species, thereby providing a new reference for further understanding bacterial diversity in hypersaline environments. This study also suggested that the diversity of bacteria isolated from Aiding Lake is largely dependent on the isolation media. Obviously, the number and diversity of bacteria isolated from different media are different in this study. Although media I and E showed the lowest recoverability at the genus level, the microorganism of *Sinobaca* genus was only isolated using medium I, and a novel strain ADL013 was found using the medium too. At the same time, the microorganism of phylum *Rhodothermaeota* was detected only using medium E. Of all the media, the most actinobacterial genera (7) were isolated from medium C. This result suggests that medium C is suitable for the isolation of actinomycetes in sediments of salt lake. In addition, the most abundant bacterial genera were obtained from medium G (Microcrystalline cellulose-sorbitol agar), this may indicate that these bacteria have a special utilization for microcrystalline cellulose, or sorbitol, or Beta-Cyclodextrin, but at present the mechanism of why medium G is suitable for the isolation of bacteria from Aiding lake is unclear. No universal medium or uniform isolation technology has been established for microbial resources around the world. Therefore, there is a need to develop novel isolation media or isolation techniques to better mine non-culturable bacterial resources.

## Supporting information

S1 Fig Geographic location of Aiding Lake. (TIF)

S1 Table The number of isolates recovered from each sediment. (DOCX)

## References

[1] Oren A. Industrial and environmental applications of halophilic microorganisms. Environ Technol 2010; 31: 825–834. 10.1080/09593330903370026 20662374

[2] Oren A. Halophilic microbial communities and their environments. Curr Opin Biotech 2015; 33: 119–124. 10.1016/j.copbio.2015.02.005 25727188

[3] Oren A. Molecular ecology of extremely halophilic Archaea and Bacteria. FEMS Microbiol Ecol 2003; 39(1): 1–7. 10.1111/j.1574-6941.2002.tb00900.x 19709178

[4] Antonio V, Rafael RLH, Cristina SP, Thane PR. Microbial diversity of hypersaline environments: a metagenomic approach. Curr Opin Microbiol 2015; 25: 80–87. 10.1016/j.mib.2015.05.002 26056770

[5] Ali N, Giti E, Mohammad AA, Mariana SC, Lucas JS, Zahra E, et al Microbial diversity in the hypersaline lake meyghan, Iran. Scientific Reports 2017; 7: 11522 10.1038/s41598-017-11585-3 28912589 PMC5599592

[6] Manel BA, Fatma K, Najwa K, Fabrice A, Najla M, Marianne Q, et al Abundance and diversity of prokaryotes in ephemeral hypersaline lake Chott El Jerid using Illumina Miseq sequencing, DGGE and qPCR assays. Extremophiles 2018; 22(5): 811–823. 10.1007/s00792-018-1040-9 30014241

[7] Nahid O, David JC, Inmaculada L, Victoria B, Fernando MC. Study of bacterial community composition and correlation of environmental variables in Rambla Salada, a hypersaline environment in south-eastern Spain. Front Microbiol 2018; 9: 1377–1383. 10.3389/fmicb.2018.01377 29977233 PMC6021518

[8] DasSsrma S, Arora P. 2001 Halophiles, p. 458–466. In Battista J. R. et al (ed.), Encyclopedia of life sciences, vol. 8 Nature Publishing Group, London, Untied Kingdom.

[9] Oren A. The bioenergetics basis for the decrease in metabolic diversity ecosystems. Hydrobiologia 2001; 466: 61–72.

[10] Meglio LD, Santos F, Gomariz M, Almansa C, Lopez C, Anton J, et al Seasonal dynamics of extremely halophilic microbial communities in three argentinian salterns. FEMS Microbiol Ecol 2016; 92(12): 184 10.1093/femsec/fiw184 27604253

[11] Oren A. Diversiyt of halophilic microorganisms: environments, phylogeny, physiology, and applications. J Ind Microbiol Biotechnol 2002; 28(1): 56–63. 10.1038/sj/jim/7000176 11938472

[12] Addis S, Anders L, Amare G, Lise O. Prokaryotic community diversity along an increasing salt gradient in a soda Ash concentration Pond. Microbiol Ecology 2016; 71(2): 326–338. 10.1007/s00248-015-0675-7 26408190

[13] Jiang HC, Dong HL, Zhang GX, Yu BS, Leah RC, Matthew WF. Microbial diversity in water and sediment of lake Chaka, an athalassohaline lake in northwestern China. Appl Environ Microbiol 2006; 72(6): 3832–3845. 10.1128/AEM.02869-05 16751487 PMC1489620

[14] Loubna T, Donald PB, Alan RH, Keith AC. Life in extreme environments: microbial diversity in Great Salt Lake, Utah. Extremophiles 2014; 18(3): 525–535. 10.1007/s00792-014-0637-x 24682608

[15] Han R, Zhang X, Liu J, Long QF, Chen LS, Liu DL, et al Microbial community structure and diversity within hypersaline Keke salt lake environments. Can J Microbiol 2017; 63(11): 895–908. 10.1139/cjm-2016-0773 28850799

[16] Baxter BK. Great Salt Lake microbiology: a historical perspective. Int Mcrobiology 2018; 21(3): 79–95. 10.1007/s10123-018-0008-z PMC6133049 30810951

[17] Guan TW, Wei B, Zhang Y, Xia ZF, Che ZM, Chen XG, et al *Actinopolyspora lacussalsi* sp. nov., *Actinopolyspora lacussalsi* sp. nov., an exteremely halophilic actinomycete isolated from a salt lake. Int J Syst Evol Microbiol 2013; 63: 3009–3013. 10.1099/ijs.0.047167-0 23396724

[18] Guan TW, Zhao K, Xiao J, Liu Y, Xia ZF, Zhang XP, et al *Brevibacterium salitolerans* sp. nov., an actinobacterium isolated from a salt lake in Xinjiang, China. Int J Syst Evol Microbiol 2010; 60: 2991–2995. 10.1099/ijs.0.020214-0 20118289

[19] Tang SK, Wang Y, Guan TW, Lee JC, Kim CJ, Li WJ. *Amycolatopsis halophila* sp. nov., a novel halophilic actinomycete isolated from a salt lake in China. Int J Syst Evol Microbiol 2010; 60(5): 1073–1078. 10.1099/ijs.0.012427-0 19666809

[20] Duangmal K, Suksaard P, Pathom-aree W, Mingma R, Matsumoto A, Takahashi Y, et al *Actinopolyspora salinaria* sp. nov., a halophilic actinomycete isolated from solar saltern soil. Int J Syst Evol Microbiol 2016; 68(4): 3506–3511. 10.1099/ijsem.0.000926 26812900

[21] Meng XL, Ming H, Huang JR, Zhang LY, Cheng LJ, Zhao ZL, et al *Paracoccus halotolerans* sp. nov., isolated from a salt lake. Int J Syst Evol Microbiol 2019: 69: 523–528. 10.1099/ijsem.0.003190 30570476

[22] Harjodh S, Manpreet K, Shweta S, Sunita M, Shekhar K, Venkata RV, et al *Salibacterium nitratireducens* sp nov., a haloalkalitolerant bacterium isolated from a water sample from Sambhar salt lake, India. Int J Syst Evol Microbiol 2018; 68: 3506–3511. 10.1099/ijsem.0.003021 30226463

[23] Yakimov M, Giuliano L, Crisafi E, Chernikova T, Timmis K, Golyshin P. Microbial community of a saline mud volcano at San Biagio-Belpasso, Mt. Etna (Italy). Environ Microbiol 2002; 4(5): 249–256. 10.1046/j.1462-2920.2002.00293.x 12030850

[24] Shirling EB, Gottlieb D. Methods for characterization of Streptomyces species. Int J Syst Bacteriol 1966; 16: 313–340. 10.1099/00207713-16-3-313

[25] Li WJ, Xu P, Schumann P, Zhang YQ, Pukall R, Xu LH, et al *Georgenia ruanii* sp. nov., a novel actinobacterium isolated from forest soil in Yunnan (China), and emended description of the genus *Georgenia*. Int J Syst Evol Microbiol 2007; 57: 1424–1428. 10.1099/ijs.0.64749-0 17625169

[26] Yoon SH, Ha SM, Kwon S, Lim J, Kim Y, Seo H, et al Introducing EzBioCloud: A taxonomically united database of 16S rRNA and whole genome assemblies. Int. J. Syst. Evol. Microbiol 2017; 67: 1613–1617. 10.1099/ijsem.0.001755 28005526 PMC5563544

[27] Tamura K, Stecher G, Peterson D, Filipski A, Kumar S. MEGA6: Molecular Evolutionary enetics Analysis Version 6.0. Mol Biol Evol 2013; 30(12): 2725–2729. 10.1093/molbev/mst197 24132122 PMC3840312

[28] Saitou N, Nei M. The neighbor-joining method: a new method for reconstructing phylogenetic tree. Mol Biol Evol 1987; 4(4): 406–425. 10.1093/oxfordjournals.molbev.a040454 3447015

[29] Kimura M. A simple method for estimating evolutionary rates of base substitutions through comparative studies of nucleotide sequence. J Mol Evol 1980; 16(2): 111–120. 10.1007/BF01731581 7463489

[30] Felsenstein J. Confidence limits on phylogenies: an approach using the bootstrap. Evolution 1985; 39: 783–789. 10.1111/j.1558-5646.1985.tb00420.x 28561359

[31] Ezaki T, Hashimoto Y, Yabuuchi E. Fluorometric deoxyribonucleic acid-deoxyribonucleic acid hybridization in microdilution wells as an alternative to membrane filter hybridization in which radioisotopes are used to determine genetic relatedness among bacterial strains. Int J Syst Bacteriol 1989; 39: 224–229.

[32] Stackebrandt E, Goebel BM. Taxonomic note: a place for DNA-DNA reassociation and 16S rRNA sequence analysis in the present species definition in bacteriology. Int J Syst Bacteriol 1994; 44: 846–849. 10.1099/00207713-44-4-846

[33] Phillips K, Frederic 3rd Z, Elizondo OR, Lowe KL. Phenotypic characterization and 16S rDNA identification of culturable non-obligate halophilic bacterial communities from a hypersaline lake, La Sal del Rey, in extreme South Texas (USA). Aquatic. Biosystems 2012; 8(1): 5–15. 10.1186/2046-9063-8-5 22480362 PMC3310331

[34] Li X, Yu YH. Biodiversity and screening of halophilic bacteria with hydrolytic and antimicrobial activities from yuncheng salt lake, China. Biologia 2015; 70(2): 151–156. 10.1515/biolog-2015-0033

[35] Rohban R, Amoozegar MA, Ventosa A. Screening and isolation of halophilic bacteria producing extracellular hydrolyses from Howz Soltan Lake, Iran. J Ind Microbiol Biot 2009; 36(3): 333–340. 10.1007/s10295-008-0500-0 19037673

[36] Wu JY, Guan TW, Jiang HC, Zhi XY, Tang SK, Dong HL, et al Diversity of actinobacterial community in saline sediments from Yunnan and Xinjiang, China. Extremophiles 2009; 13(4): 623–632. 10.1007/s00792-009-0245-3 19504229

[37] Ellis GA, Thomas CS, Chanana S, Adnani N, Szachowicz E, Braun DR, et al Brackish habitat dictates cultivable actinobacterial diversity from marine sponges. Plos One 2017; 12(7): e0176968 10.1371/journal.pone.0176968 28692665 PMC5503172

[38] Zhang GY, Cao TF, Ying JX, Yang YL, Ma LQ. Diversity and novelty of actinobacteria in Arctic marine sediments. Antonie. Van. Leeuwenhoek 2014; 105(4): 743–754. 10.1007/s10482-014-0130-7 24519808

[39] Atika M, Nasserdine S, Abdelghani Z, Florence M, Ahmed L. Isolation, taxonomy, and antagonistic properties of halophilic actinomycetes in Saharan soils of Algeria. Appl Environ Microbiol 2011; 77(18): 6710–6714. 10.1128/AEM.00326-11 21764956 PMC3187135

[40] Ronoh RC, Budambula NLM, Mwirichia RK, Boga HI. Isolation and characterization of actinobacteria from lake Magadi, Kenya. Afr J Microbiol Res 2013; 7: 4200–4206. 10.5897/AJMR2012.2296

[41] Wang Y, Tang SK, Lou K, Lee JC, Jeon CO, Xu LH, et al *Aidingimonas halophila* gen. nov., sp. nov., a moderately halophilic bacterium isolated from a salt lake. Int J Syst Evol Microbiol 2009; 59(12): 3088–3094. 10.1099/ijs.0.010264-0 19643876

[42] Wayne LG, Brenner DJ, Colwell RR, et al International Committee on Systematic Bacteriology. Report of the ad hoc committee on reconciliation of approaches to bacterial systematics. Int J Syst Bacteriol 1987; 37: 463–464.

[43] Xia ZF, Ruan JS, Huang Y, Zhang LL. *Streptomyces aidingensis* sp. nov., an actinomycete isolated from lake sediment. Int J Syst Evol Microbiol 2013; 63(9): 3204–3208. 10.1099/ijs.0.049205-0 23456804

[44] Xia ZF, Guan TW, Ruan JS, Huang Y, Zhang LL. *Longimycelium tulufanense* gen. nov., sp. nov., a filamentous actinomycete of the family Pseudonocardiaceae. Int J Syst Evol Microbiol 2013; 63(8): 2813–2818. 10.1099/ijs.0.044222-0 23315412

[45] Nikou MM, Ramezani M, Harirchi S, Makzoom S, Amoozegar MA, Shahzadeh FSA, et al Salinifilum gen. nov., with description of *Salinifilum proteinilyticum* sp nov., an extremely halophilic actinomycete isolated from Meighan wetland, Iran, and reclassification of *Saccharopolyspora aidingensis* as *Salinifilum aidingensis* comb. nov and *Saccharopolyspora ghardaiensis* as *Salinifilum ghardaiensis* comb. Nov Int J Syst Evol Microbiol 2017; 67(10): 4221–4227. 10.1099/ijsem.0.002286 28920832

[46] Guan TW, Tian L, Li EY, Tang SK, Zhang XP. *Gracilibacillus aidingensis* sp. nov., a novel moderately halophilic bacterium isolated from Aiding salt lake. Arch Microbiol 2017; 199(9): 1277–1281. 10.1007/s00203-017-1399-5 28624973

[47] Guan TW, Xiao J, Zhao K, Luo XX, Zhang XP, Zhang LL. *Halomonas xinjiangensis* sp. nov., a halotolerant bacterium isolated from a salt lake. Int J Syst Evol Microbiol 2010; 60(2): 349–352. 10.1099/ijs.0.011593-0 19651733

