# Supplementary figures and images for: Isolation and diversity of sediment bacteria in the hypersaline aiding lake, China
Source: PLoS One. 2020 Jul 10;15(7):e0236006. doi: 10.1371/journal.pone.0236006 (PMC7351256; doi:10.1371/journal.pone.0236006)

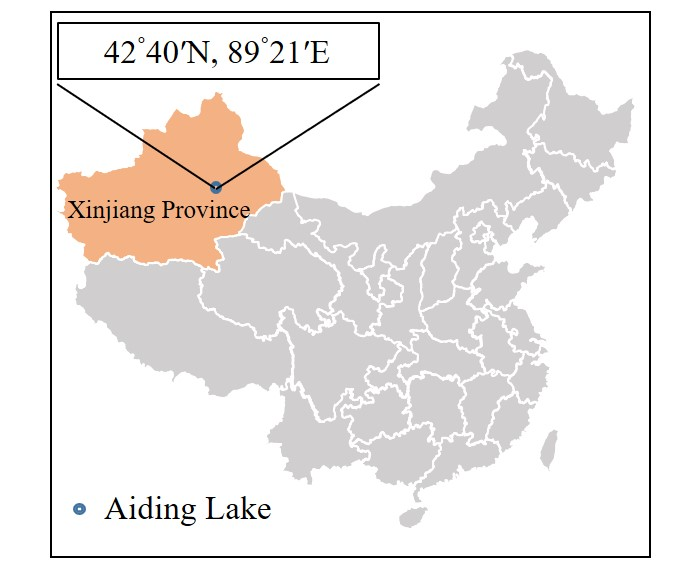

Supplement: S1 Fig — (TIF) [file pone.0236006.s001.tif]
