# Supplementary material for: Isolation and diversity of sediment bacteria in the hypersaline aiding lake, China
Source: PLoS One. 2020 Jul 10;15(7):e0236006. doi: 10.1371/journal.pone.0236006 (PMC7351256; doi:10.1371/journal.pone.0236006)
Supplement: S1 Table — (DOCX) [file pone.0236006.s002.docx]

**S1 Table. The number of isolates recovered from each sediment.**

| Genus | No. of isolates | | | Genus | No. of isolates | | |
| --- | --- | --- | --- | --- | --- | --- | --- |
|  | S1 | S2 | S3 |  | S1 | S2 | S3 |
| *Actinopolyspora* | 21 | 4 | 1 | *Nesterenkonia* | 2 | 0 | 0 |
| *Aidingimonas* | 2 | 1 | 0 | *Nitratireductor* | 0 | 0 | 3 |
| *Aliifodinibius* | 4 | 0 | 1 | *Nocardiopsis* | 2 | 4 | 11 |
| *Alteribacillus* | 0 | 1 | 8 | *Ornithinibacillus* | 0 | 1 | 0 |
| *Anaerobacillus* | 0 | 0 | 1 | *Phytoactinopolyspora* | 3 | 0 | 0 |
| *Aquibacillus* | 5 | 3 | 2 | *Piscibacillus* | 4 | 0 | 1 |
| *Aquisalimonas* | 7 | 4 | 1 | *Planococcus* | 0 | 0 | 2 |
| *Bacillus* | 9 | 3 | 3 | *Pontibacillus* | 8 | 1 | 0 |
| *Filobacillus* | 3 | 1 | 0 | *Prauserella* | 0 | 1 | 0 |
| *Glycomyces* | 0 | 2 | 0 | *Saccharomonospora* | 7 | 0 | 0 |
| *Gracilibacillus* | 24 | 6 | 3 | *Saccharopolyspora* | 5 | 0 | 0 |
| *Haloactinospora* | 3 | 0 | 0 | *Salinicoccus* | 5 | 1 | 2 |
| *Halobacillus* | 5 | 3 | 0 | *Salinifilum* | 0 | 0 | 4 |
| *Haloechinothrix* | 0 | 2 | 0 | *Sediminibacillus* | 0 | 9 | 2 |
| *Halomonas* | 21 | 4 | 17 | *Sinobaca* | 1 | 4 | 0 |
| *Jeotgalibacillus* | 1 | 3 | 0 | *Streptomyces* | 3 | 7 | 17 |
| *Kocuria* | 0 | 0 | 2 | *Virgibacillus* | 3 | 7 | 1 |
| *Longimycelium* | 0 | 4 | 1 | *Zhihengliuella* | 0 | 0 | 2 |
| *Marinactinospora* | 4 | 0 | 2 | ADL013 | 1 | 0 | 0 |
| *Marinobacter* | 3 | 5 | 0 | ADL023 | 1 | 0 | 0 |
| *Marinococcus* | 7 | 3 | 1 |  |  |  |  |
| *Micromonospora* | 0 | 2 | 3 |  |  |  |  |
| *Myceligenerans* | 0 | 2 | 0 |  |  |  |  |
