# Supplementary material for: Isolation and diversity of sediment bacteria in the hypersaline aiding lake, China
Source: PLoS One. 2020 Jul 10;15(7):e0236006. doi: 10.1371/journal.pone.0236006 (PMC7351256; doi:10.1371/journal.pone.0236006)
Supplement: Attachment — Submitted filename: response to PLOS ONE.docx [file pone.0236006.s003.docx]

**Dear Prof. Elly Welburn,**

**Thank you for your letter.**

**We have carefully read over reviewers’ advice on our paper (PONE-D-19-13013, Isolation and Diversity of Sediment Bacteria in the Hypersaline Aiding Lake, Chin). We seriously have revised them item by item based on reviewers’ comments. Obviously, we very wish to be considered for publication in PLOS ONE. So again, we resubmitted the manuscript to PLOS ONE.**

**If you need any more information concerning the manuscript, please don’t hesitate to contact with us in a reasonable amount of time by e-mail (**[**guantongweily@163.com**](mailto:guantongweily@163.com) **).**

**Best Wishes**

**Yours sincerely**

**Tong-Wei Guan**

**Comments to the Author**

1. Is the manuscript technically sound, and do the data support the conclusions?

The manuscript must describe a technically sound piece of scientific research with data that supports the conclusions. Experiments must have been conducted rigorously, with appropriate controls, replication, and sample sizes. The conclusions must be drawn appropriately based on the data presented.
**Author: Based on reviewers’ comments, we rewrote it. Please refer to the revised text for details.**

2. Has the statistical analysis been performed appropriately and rigorously?
**Author: We redid the statistical analysis and fixed some minor errors. Please refer to the revised text for details.**

3. Have the authors made all data underlying the findings in their manuscript fully available?
 **Author: Yes, we have made all data underlying the findings in the manuscript fully available.**

4. Is the manuscript presented in an intelligible fashion and written in standard English?

PLOS ONE does not copyedit accepted manuscripts, so the language in submitted articles must be clear, correct, and unambiguous. Any typographical or grammatical errors should be corrected at revision, so please note any specific errors here.
**Author: Yes, we revised the part. Please refer to the revised text for details.**

5. Review Comments to the Author
Reviewer #1: The article "Isolation and Diversity of Sediment Bacteria in the Hypersaline Aiding Lake, China" describe the isolation and analysis of diversity of some halophylic and halotorant strains from a saline lake in China. The article contains some valuable data, but the presentation if far away to be ready, the discussion is missing (just list of results from other works are given, without relationship, without talk about the objectives of the other works, the media used...) and some results are included in the discussion.

**Author: Yes, we rewrote the discussion, and the full text has been modified appropriately. Please refer to the revised text for details.**

The phyla, order, family and others taxonomic names shoud be in italics.

**Author: Yes, we changed them in the revised MS.**
If the salinity of the lake is over 30%, why not test this concentration in your media instead of 5 and 15%?

**Author: Yes, Aiding Lake is a dry salt lake. Surface layer of the soil samples have a high salinity, while the other parts have a low salinity. In addition, bacteria may tolerate a large range of salinity.**
The names of the microorganisms should be ALL revised as I found many of them that are not correctly written (i.e. Anaerobacillus the last s is missing, Actinomuaura should be Actinomadura, Streptosprangium should be Streptosporangium...)

**Author: Yes, we revised them. Please refer to the revised text for details.**
Line 44: references are given twice.

**Author: Yes, we deleted them.**
Line 54: nov instead of Nov

**Author: Yes, the nov instead of Nov in the revised MS.**
Line 200: correct to consisting

**Author: Yes, we revised it.**
Line 208: Verrucosispora was reclassified into Micromonosopora last year (Nouioui et al., 2018, Front Microbiol), so it should not be included as a different genus.

**Author: Yes, we changed it. Please refer to the revised text for details.**
Line 222: rRNA gene

**Author: Yes, we revised it.**
Line 225: could before represents

**Author: Yes, we revised it.**
Line 244: delete these

**Author: Yes, we deleted it in the revised MS.**
Table 4: will be more clear if the phylum and other groups are in line with their genera.

**Author: Yes, we revised them. At present, the phylum and other groups are in line with their genera.**
Figure one: it would be easier to have a percentage within the names directly.

**Author: Yes, thank you for your advice.**

**If we add a percentage within the names directly, it feels a bit repetitive. We think that the original diagram is ok, and don't need to add it.**
Why not include Figure 3 and Figure 4 in one adding both isolates?
**Author: Yes, we revised them on your advice.**

The manuscript by Guan et al. deals with the selective isolation and (partial) identification of a number of strains from an extreme hypersaline lake in China. The manuscript is interesting but IMHO lacks with a solid background in order to be published as it is. I further present some particular comments to support my decision.

1. Introduction (Section 1).
The abstract talks about phyla and orders but in the introduction (lines 53 to 55) examples are given from previous work regarding the isolation of microorganisms…however, the selected bacteria to which phyla and/or order belong? This can certainly cause confusion to be an unfamiliar reader and/or the authors are assuming that the reader will certainly know about the different taxonomic hierarchies.
**Author: Thanks for your comments. We added the part appropriately. Please refer to the revised text for details.**
2. Sample collection (Section 2.1) & isolation of microorganisms (section 2.2).
The authors claim to have collected 3 sediment samples from different depths (lines 73 and 74; section 2.2) but in the selective isolation procedure they only chose 3 samples. It is not clear: are the samples the same or different? Are the depths of the same or different?
**Author: Yes, we revised the part. Please refer to the revised text for details.**
3. Identification of bacteria (Section 2.3)
The authors indicate that 71 bacteria were selected for 16S rRDNA gene sequencing. However in the results section there is only one minor phylogenetic tree with less than 25 isolates. Given the current advantages of electronic pressing, there shouldn’t be a problem to include a big phylogenetic tree in the MS rather than a small, ultra-light version. This is certainly important because the title talks about “Diversity” but the resulting image (ie. phylogenetic tree) only shows a partial vision of it.
**Author: We isolated and cultured 343 representatives of 42 bacterial genera. To our knowledge, this is the first study to recover such a high diversity of culturable bacteria from salt lake sediments. The percentages of 16S rRNA gene sequence similarities (91.14% to 100%) of 71 isolates to the closest type strains are presented in Table 3. It's not necessary to build another phylogenetic tree of 71 strains. At the same time, according to your suggestion, we have made some modifications to the article.**

4. Various
There is no information on the material and methods section regarding the sediment geochemistry but it does appear in the results section.

**Author: Yes, we added the part in the 2.1 section.**
How was established that the isolates are/were halophilic? The lake has a higher NaCl content that the media employed. Or why the media employed a different (and lower) % of NaCl than the one detected in the lake?
**Author: Possible reasons for such inconsistency could be twofold: (1) bacteria may tolerate a large range of salinity; (2) surface layer of the soil samples have a high salinity, while the other parts have a low salinity.**
Table 3 is not clear. Indeed it shows the similarity of the 71 isolates that were sequenced but the final column indicates a number regarding “number of isolates”. In other words, they sequenced all the isolates or only one and then assumed -on the basis of their strategy for grouping- that other isolates are the same and then assigned a number? If that is the case, this is unsupported because the morphological traits can change very rapidly and for that purpose then a molecular grouping PCR-based technique should be included to provide more solid ground to their “grouping”.
**Author: In fact, our classification was based on the results of sequencing all strains.**

There is no doubt that this MS shows a high degree of microbial diversity but I disagree with its current form and approach. Perhaps a comparison of the 9 different media employed by the researchers would be more suitable at it is currently presented as this is also claimed in the final paragraph of the MS (lines 252 to 256).

**Author: Thanks for your comments. We revised the part. Please refer to the revised text for details.**
