# Supplementary material for: Isolation and diversity of sediment bacteria in the hypersaline aiding lake, China
Source: PLoS One. 2020 Jul 10;15(7):e0236006. doi: 10.1371/journal.pone.0236006 (PMC7351256; doi:10.1371/journal.pone.0236006)
Supplement: Attachment — Submitted filename: Response to Reviewers 2.6.docx [file pone.0236006.s004.docx]

**6 February 2020**

**Dear Agnes Pap,**

**Thank you so much for your kind help. We have replied them item by item based on your comments. According to the actual needs, we modified the relevant content submitted online. The sequences of the bacterial isolates reported in this study have been deposited to GenBank (Accession no. MK818765- MK818834, MK296404). And the Table 5 in our text have been added the revised MS (L214, L218). We hope our reply is satisfactory.**

**We sincerely hope that our paper can be published in PLOS ONE. If you need any more information concerning the manuscript, please don’t hesitate to contact with us by e-mail.**

**Best Wishes**

**Tong-Wei Guan**

PONE-D-19-20101R1

Isolation and Diversity of Sediment Bacteria in the Hypersaline Aiding Lake, China

Dr Tong-Wei Guan

Dear Dr Guan,

Thank you for submitting your manuscript entitled "Isolation and Diversity of Sediment Bacteria in the Hypersaline Aiding Lake, China" to PLOS ONE. Your manuscript files have been checked in-house but before we can proceed we need you to address the following issues:

1) In your Methods section, please provide additional information regarding the permits you obtained for the work. Please ensure you have included the full name of the authority that approved the field site access and, if no permits were required, a brief statement explaining why.

**Author: Aiding Lake is an open lake. The collection of soil samples does not require permission from any authority. So, we don't need provide additional information regarding the permits we obtained for the work.**

2) We note that you are reporting an analysis of a microarray, next-generation sequencing, or deep sequencing data set. PLOS requires that authors comply with field-specific standards for preparation, recording, and deposition of data in repositories appropriate to their field. Please upload these data to a stable, public repository (such as ArrayExpress, Gene Expression Omnibus (GEO), DNA Data Bank of Japan (DDBJ), NCBI GenBank, NCBI Sequence Read Archive, or EMBL Nucleotide Sequence Database (ENA)). In your revised cover letter, please provide the relevant accession numbers that may be used to access these data. For a full list of recommended repositories, see http://journals.plos.org/plosone/s/data-availability#loc-omics or http://journals.plos.org/plosone/s/data-availability#loc-sequencing.

**Author: The sequences of the bacterial isolates reported in this study have been deposited to GenBank (Accession no. MK818765- MK818834, MK296404), please see L113-L115.**

3)You note that your data are available within the Supporting Information files, but no such files have been included with your submission. At this time we ask that you please upload your minimal data set as a Supporting Information file, or to a public repository such as Figshare or Dryad.

**Author: According to the actual needs, we modified the relevant content submitted online. All relevant data are within the manuscript, and there are no other relevant data information files.**

4) Please ensure that you refer to Table 5 in your text as, if accepted, production will need this reference to link the reader to the Table.

**Author: Yes, we revised it in the revised MS (L214, L218).**

**Dear Luis Angel Maldonado Manjarrez, Ph.D,**

**Thank you so much for your kind help in checking throughout the manuscript. We have carefully read over your advice and seriously considered the comments of all referees. We have revised them item by item based on your and other reviewers’ comments. Please refer to the revised text for details. We sincerely hope that our paper can be published in PLOS ONE. If you need any more information concerning the manuscript, please don’t hesitate to contact with us by e-mail (**[**guantongweily@163.com**](mailto:guantongweily@163.com)**).**

**Best Wishes**

**Yours sincerely**

**Tong-Wei Guan**

Review Comments to the Author

Please use the space provided to explain your answers to the questions above. You may also include additional comments for the author, including concerns about dual publication, research ethics, or publication ethics. (Please upload your review as an attachment if it exceeds 20,000 characters)

Reviewer #1: This manuscript describes the isolation of bacteria from sediments collected from a hypersaline lake. A diversity analysis of these bacterial isolates based on the evolutionary relatedness of the 16S rRNA gene was also performed. Overall, the study presents new and interesting data with regards to the potential to access novel species and genera via a culture-based approach and highlights the value of exploring environments that are unique and underexplored. Here are some comments and recommendations for the authors:

1. Abstract: It would be helpful to the reader to understand the aim of the study at the outset and therefore should

**Author: The aim of the study had been included in the Abstract in the revised MS.**
2. Line 30: Insert the word ‘supplemented’ before ‘with 5% of 15% (w/v) NaCl’

**Author: Yes, inserted ‘supplemented’ before ‘with 5% of 15% (w/v) NaCl’ in the revised MS.**
3. Line 31: The use of the word ‘significantly’ implies statistical analyses – if any were performed, please provide details in the methodology section and provide the p-value.

**Author: Yes, deleted ‘significantly’ in line 31 in the revised MS.**
4. Line 37: Values below 20 is typically written out in full, therefore it should be ‘…two novel genera…’ and ‘…four novel species…’

**Author: Yes, we revised them in the revised MS.**
5. Line 38: Since reference is made to more than one genus, the plural, ‘genera’, should be used.

**Author: Yes, we revised it.**
6. Line 53: The sentence is ambiguous, implying that the novel species described are different forms of culture-dependent methods. To avoid the ambiguity, it is recommended that the sentence should be changed to: ‘…have been described using culture-dependent methods: Brevibacterium salitolerans…’

**Author: Yes, we revised them in the revised MS.**
7. Line 59: It is mentioned that the Turpan Basin is the hottest region in China – can you provide a temperature range to provide insight into the environment from which the samples were collected?

**Author: Yes, we provide the annual average temperature in the revised MS.**
8. Line 60: Please provide a reference to support the statements regarding temperature and salinity.

**Author: Yes, we revised them in the revised MS.**
9. Line 62: ‘bacteria diversity’ should be ‘bacterial diversity’

**Author: Yes, we revised it.**
10. Site description and sample collection: Are three sediment samples sufficient to serve as representative samples of the whole lake area? If they were not meant to be representative samples, then I would suggest changing the manuscript title to reflect this.

**Author: We think that three sediment samples are sufficient to serve as representative samples of the whole lake area.**
11. Line 77: Sediments will not dissolve in distilled water – rather state that the sediments ‘were resuspended in distilled water’.

**Author: Yes, we revised them in the revised MS.**
12. Line 86: What was the rationale behind the design of the different types of isolation media? Even though emphasis is made that these different types of media allowed for access to unique bacterial isolates, the significance of this is not really discussed or elaborated on in the manuscript. The section on this in the Discussion should therefore be expanded on. In addition, why was the particular pre-treatment method employed in this study? Do the authors feel that this also contributed towards the isolation of a wider variety of bacterial strain?

**Author: Yes, we added them in the discussion section.**
13. Lines 87-88: Stating that ‘The compositions of the nine media are shown in Table 1’ is redundant and should be deleted. Reference to the media have already been made in line 86.

**Author: Yes, we revised them.**
14. Line 90: Is there any particular reason why an incubation temperature of 37°C was used? It is 12-14 degrees higher than the temperature recorded for the areas where the sediment samples were collected.

**Author: There is no particular reason. We set the temperature based on the culture temperature of 37 for °C most bacteria.**
15. Line 111: The use of a 99% sequence identity based on 16S rRNA gene sequences as the basis for clustering into one operational taxonomic unit poses great risk of clustering isolates that are novel. It is often found in actinobacterial genera in particular where unique species share a high 16S rRNA sequence similarity, sometimes even 100%. An alternative method of dereplication should’ve been employed – either a sequence-based method (use of another taxonomic marker) or culture-based (phenotypic differences).

**Author: Yes, we revised them.**
16. Lines 119-125: Does the Geology of the region support the differences in the physicochemical properties of the three sediment samples? Is there any correlation between the isolates (genus/family) and the sediment sample isolated from?

**Author: The Geology of the region support the differences in the physicochemical properties of the three sediment samples. In addition, we add the correlation between the isolates (genus) and the sediment sample.**
17. Lines 145-148: Make sure that all mention of ‘strains’ are correct – in some instances, the singular, ‘strain’ is used instead of the plural form.

**Author: Yes, we revised them.**
18. Lines 155-156: Correct the sentence structure – ‘…suggesting that these may represent two novel genera of Bacillaceae’.

**Author: Yes, we revised them.**
19. Line 170: ’17 families’ not ’17 family’.

**Author: Yes, we revised it.**
20. Line 171-172: A statement is made here that most bacterial groups were isolated on one specific medium – this needs to be expanded on in the discussion section.

**Author: Yes, we revised them.**
21. Line 184: ‘…one novel genera’ should read ‘…one novel genus’.

**Author: Yes, we revised it.**
22. Line 195: ‘…16S rDNA…’ should be ‘…16S rRNA genes…’

**Author: Yes, we revised them.**
23. In the discussion section, mention is made of the various genera isolated from other types of salt lakes. However, different media and isolation techniques would’ve been used to that of the study reported here. Comparisons can therefore not be made among these studies. Did any of these studies report on community analysis?

**Author: Different media and isolation techniques have been used to compare the various genera isolated from other types of salt lakes. Although the comparison has certain limitation, but it still has certain reference value. We hope that this content be retained.**
24. Lines 215-216: ‘Micromonospora’ is mentioned twice.

**Author: Yes, we revised them.**
25. Lines 229-230: See comment above drawing comparisons.

**Author: Yes, we revised them.**
26. The aspect that should be highlighted in the discussion section, is the value of the different media types in accessing different genera/species. Are there any reasons why certain genera were isolated on specific media? Any correlation to the physicochemical properties of the sediment samples?

**Author: We add the correlation between the isolates (genus) and the sediment sample, and we refined the discussion section.**
27. Please make sure that the manuscript is edited for grammar and language usage. Some examples are listed above, but this is not a comprehensive evaluation.
**Author: Yes, we revised them.**
Reviewer #2: The manuscript (MS) by Guan and colleagues describes the isolation of novel bacteria from a hypersaline lake in China. The MS is interesting but IMHO contains several flaws that require attention before it can be accepted for publication as I point below.
General comments.
1. During the MS when referring to the name and/or lists of different genera, the authours sometimes use an alphabetical order and sometimes they don’t. They should be more homogenous through the entire MS. For instance, the abstract is not in alphabetical order but in lines 172 to 174 the names of the genera mentioned are precisely in alphabetical oder. It is nt critical to use one or another but homogeneity would definitively be preferred.

**Author: Yes, we revised them. The name and/or lists of different genera mentioned are precisely in alphabetical oder in the revised MS.**
2. It should be clear for the authors that not every reader ifs familiar with the geography of China. Hence, I would stongly suggest that for the final versión of the MS, the authours include a map of China showing the exact location of the lake. This can be referred in section 2.1 Site descriptions and sample collection. In addition, is there any reference for the sentence “The high salinity, low nutrients level, dry climate, and high UV intensity makes it an extreme environment.” (Lines 69-70). If there is any, then it should be added to the MS.

**Author:** **We added a map of China showing the exact location of the lake.**

**And we deleted the sentence “The high salinity, low nutrients level, dry climate, and high UV intensity makes it an extreme environment.”**
3. Section 2.2. There is a bunch an wide range of seletive isolation media and the authors claim that they used 9 (line 86, Table 1). However when checking Table 1, all but one (ie. eight) are media “From this study”. This is intriguing because when other academics might check the MS in it final form they can argue why and/or how these eight media were chosen since this is not included nor mentioned anywhere in the current form of the MS. Therefore, I believe that the authours should add a line and/or sentence and/or paragraph indicating why and how these media was chosen for the study.

**Author: Yes, we added them in the discussion section.**
4. It is not clear to me why there are only 70 sequences deposited in GenBank (MK818765-MK818834 = 69 sequences plus MK296404 = 70) if the authors mentioned that they isolated 343 bacteria. That is only a mere 20%. If the identifiction of the bacteria is purely based on 16S rDNA gene sequencing then I think they should, at least, submit 50-60% of their sequences to GenBank and not saying that there is diversity but not providing the corresponding sequences for their isolates.

**Author: 343 isolated strains of bacteria showed that many of them had exactly the same 16S rRNA sequence. When we removed the duplicates, only 71 strains showed inconsistent sequences. Sequences of the exact same strain came from the same salt lake, so we didn't think it was necessary to submit these duplicate sequences.**
5. Section 3.1. According to the results from the authors, they sampled in three different places and provide the information in table 2. In the MS they also indicate that the conditions of each sample are different and this is very well exemplified by the high bacterial diversity. Since the authors employed 9 different media in the study, then I would strongly suggest a table indicating the number of isolates recovered from each sediment. Perhaps there is one sediment that showed a higher degree of diversity over the others. These would also help the authors to decide how many other sequences can be submitted to GenBank rather than submitting all of them as I suggested for point number 4.

**Author: We have perfected the corresponding part. And we add a table indicating the number of isolates recovered from each sediment (Supplementary table 1).**

6. Also, how did the authors checked for “duplicating strains”? Again, is this only base don the 16S rDNA gene sequencing of the isolates? This is indicted in Section 3.2, lines 127-128.

**Author: According to the analysis of sequencing results, many of isolated strains had exactly the same 16S rRNA sequence. 343 isolated strains belong to 71 different bacterial species after merging the duplicated strains (Table 3).**
7. I am not convinced by the fact that a 97% similarity (less) refers to a different species. There are examples of either a novel species or a putative novel genera. This should be handle with care. If they haven’t done so, then I suggest that the authors also genome sequence some of the most interesting isolates (ie the ones that are the most different ones acoording to EZBiocloud) and then make or at least mention DNA-DNA in silico (available from the DSMZ website) to highlight this point. However I would strongly object adding the full genome sequencing information to this MS or it would definitively become a never ending story.

**Author: Yes, we revised them.**
8. Section 3.2 lines 143 – 148. If the reader is not familiar with families and genera, these lines become extremely confusing because there is n indication of which genera is either Actinobacteria, Firmicutes, Proteobacteria or Rhodothermaeota. This is definitively a very interesting paragraph because it certainly highlights the diversity found in the study but there is no separation between any phyla thus making it difficult and confusing.

**Author: We described in detail the interrelationships among the phylum(Actinobacteria, Firmicutes, Proteobacteria or Rhodothermaeota) , order, family, or genera in table 4.**

Due to this number of general comments, then I find the Discussion section hard to follow. Besides, the English should definitively be improved in order to exploit the full potential of this MS as there are not too many studies on extreme lake environments.
I should mention that I find the MS very interesting but I am afraid in its present form I would be against its publication mostly because of its current form. It can certainly hav a higher impact if it is re-arranged/presented in a better way and hopefully the authors would be willing to incorporate all of the comments.
Minor comments.
1. Section 2.3, line 97. I believe reference 25 must be the one for the EZBiocloud website. Apparently some references are misplaced causing confusion because also reference 26 is not the one for the sentence.

**Author: Yes, we revised them.**
2. Section 2.3, line 102. How many base pairs were obtained for the 16S rDNA gene sequence? This is not indicated or are the authors assuming that anyone will check the size of the sequences deposited in the GenBank database?

**Author: Yes, we added them.**
3. Line 231, shouldn’t it be “are novel” instead of “as novel”?

**Author: Yes, we revised it.**
